# (3*Z*,5*E*)-2-Amino-4,6-bis(pyridin-3-yl)hepta-1,3,5-triene-1,1,3-tricarbo­nitrile

**DOI:** 10.1107/S2414314620012468

**Published:** 2020-09-15

**Authors:** Changting Cai, Bicheng Lin, Wenxuan Wu, Qingwei Zhu

**Affiliations:** a Department of Chemistry, Anhui University, Hefei, Anhui 230039, People’s Republic of China; University of Aberdeen, Scotland

**Keywords:** crystal structure, pyridine ring, di­cyano

## Abstract

In the title compound, C_20_H_14_N_6_, the dihedral angle between the pyridine rings is 37.98 (7)°. In the crystal, N—H⋯N hydrogen bonds link the mol­ecules into (10  ) sheets.

## Abstract 2

Hydrogen-bonded sheets occur in the centrosymmetric crystal structure of the title compound.

## Graphical abstract

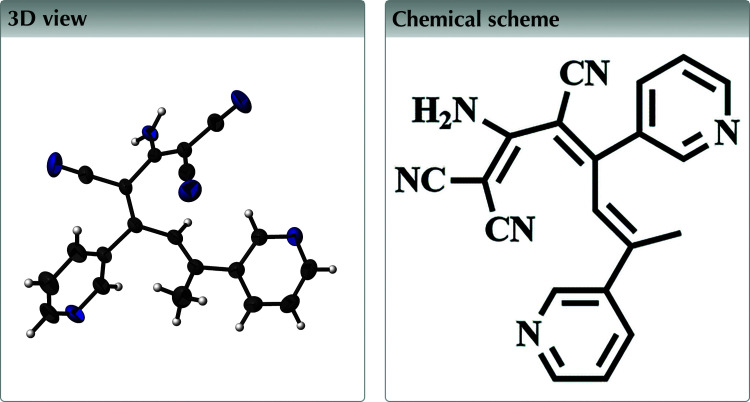

## Structure description

Nitro­gen-containing heterocyclic mol­ecular materials are widely used in optoelectronic materials (*e.g.*, Gu *et al.*, 2017 ▸) because of their donor–acceptor conjugation systems and good photophysical properties. As part of our studies in this area, we now describe the synthesis and structure of the centrosymmetric title compound in which electron-withdrawing cyanide groups have been introduced into the conjugated system.

**Figure 1:**
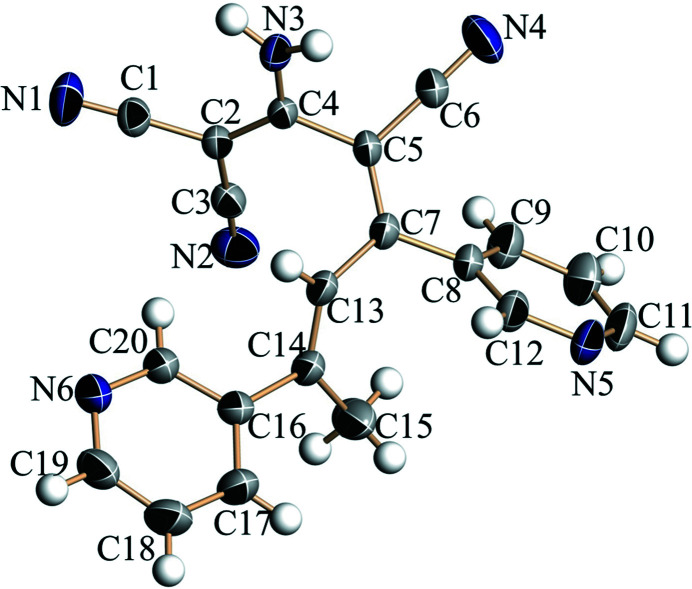
The mol­ecular structure of the title compound, with displacement ellipsoids drawn at the 30% probability level.

**Figure 2:**
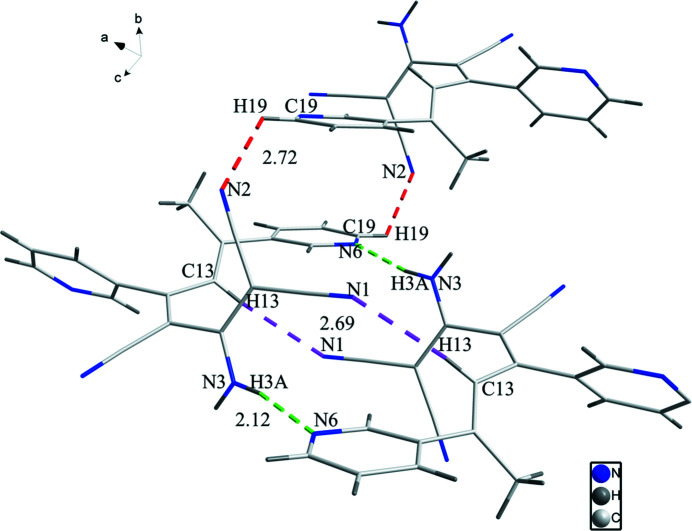
Partial packing diagram of the title compound showing selected inter­molecular contacts.

The crystal structure (Fig. 1 ▸) shows that the dihedral angle between the pyridine ring planes is 37.98 (7)°. In the crystal, inversion dimers linked by pairs of N3—H3*A*⋯N6 hydrogen bonds (Table 1 ▸, Fig. 2 ▸) generate 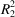 (20) loops. The dimers are linked into (10 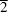 ) sheets by N3—H3*B*⋯N5 hydrogen bonds. It is noteworthy that both acceptor atoms are parts of the pyridine rings.

## Synthesis and crystallization

3-Acetyl­pyridine (12.0 g, 0.010 mol) and ammonium acetate (6.6 g, 0.01 mol) were dissolved in 200 ml of ethanol. Malono­nitrile (6.6 g, 0.01 mol), was added and the mixture was heated to 318 K. When the color of the solution changed from colorless to orange–red, 10 drops of glacial acetic acid were added to the system and 14 g (yield 83%) of yellow powder was recovered. Yellow block-shaped crystals suitable for X-ray analysis were obtained by recrystallization from ethanol solution.

## Refinement

Crystal data, data collection and structure refinement details are summarized in Table 2 ▸.

## Supplementary Material

Crystal structure: contains datablock(s) I. DOI: 10.1107/S2414314620012468/hb4363sup1.cif

Structure factors: contains datablock(s) I. DOI: 10.1107/S2414314620012468/hb4363Isup3.hkl

CCDC reference: 2020071

Additional supporting information:  crystallographic information; 3D view; checkCIF report

## Figures and Tables

**Table 1:** Hydrogen-bond geometry (Å, °)

*D*—H⋯*A*	*D*—H	H⋯*A*	*D*⋯*A*	*D*—H⋯*A*
N3—H3*A*⋯N6^i^	0.89	2.12	2.9903 (17)	168
N3—H3*B*⋯N5^ii^	0.88	2.10	2.9372 (16)	159

Symmetry codes: (i) 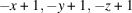 ; (ii) 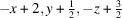 .

**Table 2:** Experimental details

Crystal data	
Chemical formula	C_20_H_14_N_6_
*M*_r_	338.37
Crystal system, space group	Monoclinic, *P*2_1_/*c*
Temperature (K)	296
*a*, *b*, *c* (Å)	8.4636 (13), 9.8402 (15), 22.701 (3)
β (°)	105.917 (5)
*V* (Å^3^)	1818.1 (5)
*Z*	4
Radiation type	Mo *K*α
μ (mm^−1^)	0.08
Crystal size (mm)	0.30 × 0.20 × 0.10

Data collection	
Diffractometer	Bruker APEXII CCD
Absorption correction	Multi-scan (*SADABS*; Bruker, 2013 ▸)
*T*_min_, *T*_max_	0.593, 0.746
No. of measured, independent and observed [*I* > 2σ(*I*)] reflections	14113, 3912, 3314
*R*_int_	0.043
(sin θ/λ)_max_ (Å^−1^)	0.650

Refinement	
*R*[*F* ^2^ > 2σ(*F* ^2^)], *wR*(*F* ^2^), *S*	0.042, 0.114, 1.03
No. of reflections	3912
No. of parameters	236
H-atom treatment	H-atom parameters constrained
Δρ_max_, Δρ_min_ (e Å^−3^)	0.24, −0.19

Computer programs: *APEX2* and *SAINT* (Bruker, 2013 ▸), *olex2.solve* (Bourhis *et al.*, 2015 ▸), *SHELXL* (Sheldrick, 2015 ▸) and *OLEX2* (Dolomanov *et al.*, 2009 ▸).

## full crystallographic data

### Crystal data

**Table:**

C_20_H_14_N_6_	*F*(000) = 704
*M**_r_* = 338.37	*D*_x_ = 1.236 Mg m^−^^3^
Monoclinic, *P*2_1_/*c*	Mo *K*α radiation, λ = 0.71073 Å
*a* = 8.4636 (13) Å	Cell parameters from 7944 reflections
*b* = 9.8402 (15) Å	θ = 2.3–27.2°
*c* = 22.701 (3) Å	µ = 0.08 mm^−^^1^
β = 105.917 (5)°	*T* = 296 K
*V* = 1818.1 (5) Å^3^	Block, yellow
*Z* = 4	0.30 × 0.20 × 0.10 mm

### Data collection

**Table:**

Bruker APEXII CCD diffractometer	3314 reflections with *I* > 2σ(*I*)
ω scans	*R*_int_ = 0.043
Absorption correction: multi-scan (SADABS; Bruker, 2013)	θ_max_ = 27.5°, θ_min_ = 1.9°
*T*_min_ = 0.593, *T*_max_ = 0.746	*h* = −11→10
14113 measured reflections	*k* = −12→12
3912 independent reflections	*l* = −28→28

### Refinement

**Table:**

Refinement on *F*^2^	Primary atom site location: structure-invariant direct methods
Least-squares matrix: full	Hydrogen site location: mixed
*R*[*F*^2^ > 2σ(*F*^2^)] = 0.042	H-atom parameters constrained
*wR*(*F*^2^) = 0.114	*w* = 1/[σ^2^(*F*_o_^2^) + (0.0519*P*)^2^ + 0.4095*P*] where *P* = (*F*_o_^2^ + 2*F*_c_^2^)/3
*S* = 1.03	(Δ/σ)_max_ = 0.001
3912 reflections	Δρ_max_ = 0.24 e Å^−^^3^
236 parameters	Δρ_min_ = −0.18 e Å^−^^3^
0 restraints	

### Special details

**Table:**

Geometry. All esds (except the esd in the dihedral angle between two l.s. planes) are estimated using the full covariance matrix. The cell esds are taken into account individually in the estimation of esds in distances, angles and torsion angles; correlations between esds in cell parameters are only used when they are defined by crystal symmetry. An approximate (isotropic) treatment of cell esds is used for estimating esds involving l.s. planes.
Refinement. The C-bound H atoms were geometrically placed (C—H = 0.93–0.96 Å) and refined as riding atoms. The N-bound H atoms were located in difference maps and refined as riding atoms in their as-found relative positions.

### Fractional atomic coordinates and isotropic or equivalent isotropic displacement parameters (Å^2^)

**Table:**

	*x*	*y*	*z*	*U*_iso_*/*U*_eq_	
N1	0.56958 (17)	0.53555 (15)	0.42295 (5)	0.0603 (4)	
N2	0.94585 (16)	0.23652 (13)	0.49739 (6)	0.0549 (3)	
N3	0.79713 (13)	0.65640 (10)	0.57511 (5)	0.0390 (2)	
H3A	0.741712	0.707730	0.544186	0.047*	
H3B	0.814112	0.687880	0.612586	0.047*	
N4	1.21602 (16)	0.64094 (15)	0.67229 (7)	0.0643 (4)	
N5	1.19544 (15)	0.19840 (13)	0.79606 (5)	0.0520 (3)	
N6	0.35370 (14)	0.13699 (12)	0.52035 (6)	0.0500 (3)	
C1	0.67660 (15)	0.50431 (13)	0.46360 (5)	0.0388 (3)	
C2	0.80838 (14)	0.46093 (12)	0.51396 (5)	0.0325 (2)	
C3	0.88674 (15)	0.33700 (13)	0.50564 (5)	0.0375 (3)	
C4	0.85396 (13)	0.53460 (11)	0.56833 (5)	0.0313 (2)	
C5	0.97970 (14)	0.47672 (12)	0.62215 (5)	0.0337 (3)	
C6	1.11384 (15)	0.56647 (14)	0.64999 (6)	0.0410 (3)	
C7	0.96757 (14)	0.35134 (12)	0.64586 (5)	0.0343 (3)	
C8	1.10641 (15)	0.29486 (13)	0.69512 (5)	0.0369 (3)	
C9	1.26451 (16)	0.28301 (16)	0.68862 (6)	0.0492 (3)	
H9	1.288538	0.312862	0.653171	0.059*	
C10	1.38551 (18)	0.22577 (18)	0.73606 (7)	0.0580 (4)	
H10	1.491932	0.215363	0.732728	0.070*	
C11	1.34588 (19)	0.18459 (16)	0.78815 (6)	0.0556 (4)	
H11	1.427618	0.145165	0.819473	0.067*	
C12	1.07878 (16)	0.25103 (14)	0.74965 (6)	0.0432 (3)	
H12	0.973100	0.258789	0.754047	0.052*	
C13	0.81217 (14)	0.27732 (13)	0.62629 (5)	0.0366 (3)	
H13	0.718338	0.331166	0.615438	0.044*	
C14	0.78551 (15)	0.14270 (13)	0.62173 (6)	0.0410 (3)	
C15	0.9134 (2)	0.03265 (16)	0.63531 (9)	0.0667 (5)	
H15A	1.019436	0.071646	0.638498	0.100*	
H15B	0.888321	−0.033182	0.602860	0.100*	
H15C	0.914337	−0.010841	0.673256	0.100*	
C16	0.61170 (16)	0.09860 (13)	0.59646 (6)	0.0415 (3)	
C17	0.53955 (19)	−0.00536 (16)	0.62118 (7)	0.0566 (4)	
H17	0.601159	−0.054456	0.654636	0.068*	
C18	0.3753 (2)	−0.03560 (16)	0.59575 (8)	0.0622 (4)	
H18	0.325133	−0.104969	0.611867	0.075*	
C19	0.28733 (18)	0.03862 (15)	0.54626 (8)	0.0557 (4)	
H19	0.176290	0.019311	0.530079	0.067*	
C20	0.51241 (16)	0.16395 (14)	0.54536 (6)	0.0440 (3)	
H20	0.560161	0.231478	0.527256	0.053*	

### Atomic displacement parameters (Å^2^)

**Table:**

	*U*^11^	*U*^22^	*U*^33^	*U*^12^	*U*^13^	*U*^23^
N1	0.0603 (8)	0.0704 (9)	0.0382 (6)	0.0141 (7)	−0.0065 (6)	−0.0033 (6)
N2	0.0563 (7)	0.0427 (7)	0.0675 (8)	0.0025 (6)	0.0203 (6)	−0.0034 (6)
N3	0.0455 (6)	0.0360 (5)	0.0295 (5)	0.0030 (4)	0.0003 (4)	0.0003 (4)
N4	0.0456 (7)	0.0640 (9)	0.0725 (9)	−0.0165 (6)	−0.0019 (6)	−0.0138 (7)
N5	0.0559 (7)	0.0589 (7)	0.0328 (5)	0.0047 (6)	−0.0018 (5)	0.0094 (5)
N6	0.0394 (6)	0.0452 (6)	0.0561 (7)	−0.0040 (5)	−0.0027 (5)	0.0045 (5)
C1	0.0417 (7)	0.0415 (7)	0.0301 (6)	−0.0004 (5)	0.0047 (5)	−0.0034 (5)
C2	0.0317 (6)	0.0340 (6)	0.0290 (5)	−0.0029 (5)	0.0038 (4)	0.0014 (4)
C3	0.0359 (6)	0.0386 (7)	0.0367 (6)	−0.0054 (5)	0.0076 (5)	0.0005 (5)
C4	0.0294 (5)	0.0324 (6)	0.0294 (5)	−0.0051 (4)	0.0033 (4)	0.0038 (4)
C5	0.0287 (6)	0.0379 (6)	0.0297 (5)	−0.0033 (5)	−0.0002 (4)	0.0001 (4)
C6	0.0346 (6)	0.0438 (7)	0.0390 (6)	−0.0025 (5)	0.0008 (5)	0.0001 (5)
C7	0.0319 (6)	0.0384 (6)	0.0287 (5)	0.0009 (5)	0.0020 (4)	0.0035 (5)
C8	0.0340 (6)	0.0392 (6)	0.0316 (6)	−0.0002 (5)	−0.0008 (5)	0.0038 (5)
C9	0.0396 (7)	0.0625 (9)	0.0423 (7)	0.0063 (6)	0.0059 (6)	0.0091 (6)
C10	0.0381 (7)	0.0731 (10)	0.0559 (9)	0.0140 (7)	0.0014 (6)	0.0050 (7)
C11	0.0521 (8)	0.0585 (9)	0.0422 (7)	0.0135 (7)	−0.0105 (6)	0.0059 (6)
C12	0.0404 (7)	0.0509 (8)	0.0337 (6)	0.0014 (6)	0.0025 (5)	0.0062 (5)
C13	0.0317 (6)	0.0393 (6)	0.0343 (6)	0.0004 (5)	0.0015 (5)	0.0092 (5)
C14	0.0362 (6)	0.0399 (7)	0.0412 (6)	−0.0013 (5)	0.0007 (5)	0.0100 (5)
C15	0.0485 (9)	0.0434 (8)	0.0924 (12)	0.0043 (7)	−0.0072 (8)	0.0069 (8)
C16	0.0387 (7)	0.0349 (6)	0.0461 (7)	−0.0031 (5)	0.0036 (5)	0.0055 (5)
C17	0.0525 (8)	0.0470 (8)	0.0626 (9)	−0.0076 (7)	0.0026 (7)	0.0190 (7)
C18	0.0535 (9)	0.0503 (9)	0.0794 (11)	−0.0157 (7)	0.0123 (8)	0.0137 (8)
C19	0.0400 (7)	0.0483 (8)	0.0722 (10)	−0.0092 (6)	0.0043 (7)	−0.0005 (7)
C20	0.0396 (7)	0.0400 (7)	0.0466 (7)	−0.0050 (5)	0.0018 (5)	0.0064 (6)

### Geometric parameters (Å, º)

**Table:**

N1—C1	1.1438 (16)	C9—C10	1.3859 (19)
N2—C3	1.1465 (17)	C10—H10	0.9300
N3—H3A	0.8873	C10—C11	1.376 (2)
N3—H3B	0.8795	C11—H11	0.9300
N3—C4	1.3161 (15)	C12—H12	0.9300
N4—C6	1.1407 (18)	C13—H13	0.9300
N5—C11	1.341 (2)	C13—C14	1.3430 (18)
N5—C12	1.3352 (16)	C14—C15	1.502 (2)
N6—C19	1.3341 (19)	C14—C16	1.4894 (18)
N6—C20	1.3337 (17)	C15—H15A	0.9600
C1—C2	1.4261 (16)	C15—H15B	0.9600
C2—C3	1.4253 (17)	C15—H15C	0.9600
C2—C4	1.3915 (16)	C16—C17	1.3865 (19)
C4—C5	1.4956 (15)	C16—C20	1.3895 (18)
C5—C6	1.4403 (17)	C17—H17	0.9300
C5—C7	1.3611 (17)	C17—C18	1.384 (2)
C7—C8	1.4898 (15)	C18—H18	0.9300
C7—C13	1.4619 (16)	C18—C19	1.375 (2)
C8—C9	1.3908 (18)	C19—H19	0.9300
C8—C12	1.3898 (17)	C20—H20	0.9300
C9—H9	0.9300		
			
H3A—N3—H3B	118.4	C10—C11—H11	118.3
C4—N3—H3A	123.8	N5—C12—C8	123.68 (13)
C4—N3—H3B	117.8	N5—C12—H12	118.2
C12—N5—C11	117.23 (12)	C8—C12—H12	118.2
C20—N6—C19	116.96 (12)	C7—C13—H13	115.4
N1—C1—C2	178.16 (15)	C14—C13—C7	129.28 (12)
C3—C2—C1	116.10 (10)	C14—C13—H13	115.4
C4—C2—C1	121.38 (11)	C13—C14—C15	126.78 (12)
C4—C2—C3	122.50 (10)	C13—C14—C16	116.38 (11)
N2—C3—C2	177.90 (14)	C16—C14—C15	116.69 (12)
N3—C4—C2	123.83 (10)	C14—C15—H15A	109.5
N3—C4—C5	116.89 (10)	C14—C15—H15B	109.5
C2—C4—C5	119.23 (10)	C14—C15—H15C	109.5
C6—C5—C4	115.06 (10)	H15A—C15—H15B	109.5
C7—C5—C4	123.66 (10)	H15A—C15—H15C	109.5
C7—C5—C6	121.21 (10)	H15B—C15—H15C	109.5
N4—C6—C5	177.52 (15)	C17—C16—C14	123.86 (12)
C5—C7—C8	120.36 (10)	C17—C16—C20	116.53 (12)
C5—C7—C13	119.38 (10)	C20—C16—C14	119.61 (11)
C13—C7—C8	120.07 (10)	C16—C17—H17	120.2
C9—C8—C7	122.37 (11)	C18—C17—C16	119.57 (13)
C12—C8—C7	119.57 (11)	C18—C17—H17	120.2
C12—C8—C9	118.05 (11)	C17—C18—H18	120.5
C8—C9—H9	120.7	C19—C18—C17	118.91 (14)
C10—C9—C8	118.62 (13)	C19—C18—H18	120.5
C10—C9—H9	120.7	N6—C19—C18	123.14 (13)
C9—C10—H10	120.5	N6—C19—H19	118.4
C11—C10—C9	119.03 (14)	C18—C19—H19	118.4
C11—C10—H10	120.5	N6—C20—C16	124.84 (12)
N5—C11—C10	123.35 (12)	N6—C20—H20	117.6
N5—C11—H11	118.3	C16—C20—H20	117.6
			
N3—C4—C5—C6	−48.83 (14)	C8—C9—C10—C11	−0.9 (2)
N3—C4—C5—C7	128.18 (13)	C9—C8—C12—N5	0.1 (2)
C1—C2—C4—N3	−9.54 (18)	C9—C10—C11—N5	−0.9 (3)
C1—C2—C4—C5	173.02 (11)	C11—N5—C12—C8	−1.8 (2)
C2—C4—C5—C6	128.78 (12)	C12—N5—C11—C10	2.2 (2)
C2—C4—C5—C7	−54.20 (16)	C12—C8—C9—C10	1.3 (2)
C3—C2—C4—N3	172.02 (11)	C13—C7—C8—C9	129.94 (14)
C3—C2—C4—C5	−5.42 (17)	C13—C7—C8—C12	−49.56 (17)
C4—C5—C7—C8	173.26 (11)	C13—C14—C16—C17	−135.69 (15)
C4—C5—C7—C13	−11.86 (18)	C13—C14—C16—C20	43.89 (18)
C5—C7—C8—C9	−55.22 (18)	C14—C16—C17—C18	177.62 (15)
C5—C7—C8—C12	125.28 (13)	C14—C16—C20—N6	−177.12 (14)
C5—C7—C13—C14	149.68 (13)	C15—C14—C16—C17	48.4 (2)
C6—C5—C7—C8	−9.90 (18)	C15—C14—C16—C20	−132.01 (15)
C6—C5—C7—C13	164.97 (11)	C16—C17—C18—C19	0.1 (3)
C7—C8—C9—C10	−178.20 (13)	C17—C16—C20—N6	2.5 (2)
C7—C8—C12—N5	179.59 (13)	C17—C18—C19—N6	1.6 (3)
C7—C13—C14—C15	−0.9 (2)	C19—N6—C20—C16	−0.9 (2)
C7—C13—C14—C16	−176.34 (11)	C20—N6—C19—C18	−1.2 (2)
C8—C7—C13—C14	−35.42 (19)	C20—C16—C17—C18	−2.0 (2)

### Hydrogen-bond geometry (Å, º)

**Table:**

*D*—H···*A*	*D*—H	H···*A*	*D*···*A*	*D*—H···*A*
N3—H3*A*···N6^i^	0.89	2.12	2.9903 (17)	168
N3—H3*B*···N5^ii^	0.88	2.10	2.9372 (16)	159

Symmetry codes: (i) −*x*+1, −*y*+1, −*z*+1; (ii) −*x*+2, *y*+1/2, −*z*+3/2.
